# Major depressive disorder and access to health services among people who use illicit drugs in Vancouver, Canada

**DOI:** 10.1186/s13011-018-0142-9

**Published:** 2018-01-19

**Authors:** Tara Beaulieu, Lianping Ti, M.-J. Milloy, Ekaterina Nosova, Evan Wood, Kanna Hayashi

**Affiliations:** 1 British Columbia Centre for Excellence in HIV/AIDS, St. Paul’s Hospital, 608-1081 Burrard Street, Vancouver, BC V6Z 1Y6 Canada; 2 British Columbia Centre on Substance Use, St. Paul’s Hospital, 1081 Burrard Street, Vancouver, BC V6Z 1Y6 Canada; 3 Faculty of Health Sciences, Simon Fraser University, Blusson Hall, 8888 University Drive, Burnaby, BC V5A 1S6 Canada; 4 Department of Medicine, University of British Columbia, 2775 Laurel Street, Vancouver, BC V5Z 1M9 Canada

**Keywords:** Depression, Access to care, People who use illicit drugs, Canada

## Abstract

**Background:**

People who use illicit drugs (PWUD) are commonly diagnosed with major depressive disorder (MDD). However, little is known about whether PWUD living with MDD experience additional barriers to accessing health services compared to those without MDD. We sought to identify whether MDD symptoms were associated with perceived barriers to accessing health services among people who use illicit drugs (PWUD) in Vancouver, Canada.

**Methods:**

Data were collected through prospective cohorts of PWUD in Vancouver, Canada between 2005 and 2016. Using multiple logistic regression, we examined the relationship between MDD symptoms, defined as a Centre for Epidemiologic Studies Depression (CES-D) scale total score of ≥16, and barriers to access health services. We also used descriptive statistics to examine common barriers among participants who reported any barriers.

**Results:**

Among a total of 1529 PWUD, including 521 (34.1%) females, 415 (27.1%) reported barriers to accessing health services, and 956 (62.5%) reported MDD symptoms at baseline. In multiple logistic regression analyses, after adjusting for a range of potential confounders, MDD symptoms (adjusted odds ratio [AOR] = 1.40; 95% confidence interval [CI]: 1.03–1.92) were positively and significantly associated with barriers to accessing health services. Among those who reported MDD symptoms and barriers to access, commonly reported barriers included: long wait lists/times (38.1%); and treated poorly by health care professionals (30.0%).

**Conclusion:**

These findings show that the likelihood of experiencing barriers to accessing health services was higher among PWUD with MDD symptoms compared to their counterparts. Policies and interventions tailored to address these barriers are urgently needed for this subpopulation of PWUD.

## Background

The extent of overlap between major depressive disorder (MDD) and substance use disorder (SUD) is compelling, with an estimated lifetime prevalence of co-occurrence ranging from 27 to 40% in persons with MDD [1]. There exists notable empirical evidence to shed light on the possible mechanisms underlying SUD-MDD co-occurrence, with the simplest explanation being a bidirectional causal relationship (whether direct or indirect). Other explanations tend to reflect two paths of derivation: genetic, developmental and environmental factors (e.g., socioeconomic marginalization, early life trauma and a disruptive family environment); or the self-medication hypotheses (i.e., use of illicit drugs to alleviate MDD symptoms) [1–4]. No single or consistent pattern appears to underlie co-occurrence, which lends evidence to suggest that multiple pathways of association may be acting simultaneously.

A growing body of literature has indicated that SUD-MDD co-occurrence causes a significant burden to the individual, their families, and society. For instance, this comorbidity is often associated with a greater severity of symptoms, poorer treatment response and enhanced risk for SUD and MDD recurrence [5, 6]. SUD-MDD co-occurrence also yields greater social and personal impairments (e.g., interpersonal concerns and reduced occupational functioning), and enhanced risk for comorbid psychiatric conditions, particularly posttraumatic stress disorder (PTSD) and generalized anxiety disorder (GAD) [1]. Moreover, a much higher risk of suicidal ideation and attempt has been evident among this subpopulation [1, 7]. Finally, evidence suggests that there are broader implications in the context of public health and healthcare costs. For example, SUD-MDD co-occurrence often leads to higher rates of acute service utilization (e.g., emergency department and inpatient healthcare utilization) - contributing to higher costs [8, 9].

Although there are effective pharmacological and psychotherapeutic options to treat MDD, their efficaciousness is sometimes not well understood among persons with SUD (e.g., when administered concurrently with opioid agonist therapy medications such as methadone or buprenorphine) [10]. Moreover, some prescription drugs have been found to significantly increase harms (e.g., completed suicide) [11]. Additionally, while well-designed psychotherapies and pharmacological treatments for SUD exist, therapeutic efficacy and safety may differ in the presence of MDD co-occurrence [12]. Studies which examine effective treatments for SUD-MDD co-occurrence are sparse and sometimes methodologically, open to doubt. What is clear is that access to care remains disproportionately low among individuals with SUD-MDD co-occurrence [13]. A growing body of literature has explored barriers to SUD and MDD care separately [14–18], and treatment barriers among individuals with SUD and co-occurring mental illness in a broader sense [19]. However, there is a dearth of literature on barriers to care among individuals with SUD-MDD co-occurrence [20]. Therefore, we sought to identify whether MDD symptoms were associated with perceived barriers to accessing health services among people who use illicit drugs (PWUD) in Vancouver, Canada.

## Methods

### Study design and population

Data for this study were drawn from the Vancouver Injection Drug Users Study (VIDUS), and the AIDS Care Cohort to evaluate Exposure to Survival Services (ACCESS), two prospective cohorts involving PWUD in Vancouver, Canada. The methods for these studies have been described elsewhere [21, 22]. To be eligible for VIDUS, participants must be ≥18 years of age, HIV-seronegative and report injection drug use at least one month prior to enrollment. ACCESS participants must be ≥18 years of age, HIV-seropositive and report using an illicit drug (other than or in addition to cannabis) in the month prior to enrollment. Recruitment was conducted through self-referral and street outreach.

The data collection instruments and procedures were harmonized across the two cohorts to allow for pooled analyses. At baseline and semi-annually, participants completed an interviewer-administered questionnaire that elicited information on socio-demographic characteristics, drug use patterns, access to healthcare, and other relevant exposures and outcomes. Additionally, at each visit, participants provided blood samples for HIV and HCV serologic tests and HIV disease monitoring as appropriate. A $30 (CAD) honorarium was offered to participants upon completion of each study visit. The cohorts have received ethical approval by the University of British Columbia/Providence Health Care Research Ethics Board.

### Study sample

For the present analysis, the sample was restricted to those who: 1) completed a baseline between December 2005 and May 2016; and 2) completed at least one follow-up visit directly after baseline to allow for lagged analyses. For those that did not have a Centre for Epidemiologic Studies Depression (CES-D) score at baseline but had one during follow-up, the follow-up was included as the baseline (if at least one subsequent follow-up visit occurred).

### Variable selection

The primary outcome of interest was having experienced barriers to accessing health services in the last six months, dichotomized as any barriers vs. none. Possible barriers included: 1) Limited hours of operation; 2) Long wait lists/times; 3) Didn’t know where to go; 4) Language barrier; 5) Jail/detention/prison; 6) Was treated poorly by health care professionals; 7) Difficulty keeping appointments; 8) Restricted to one physician; 9) No Personal Health Number (PHN); 10) Other (barriers unaccounted for were grouped together for analysis); 11) No barriers, at baseline. The primary explanatory variable was having depressive symptoms, defined as a Centre for Epidemiologic Studies Depression (CES-D) summed score of ≥16. Our analysis utilized this cut-point as MDD has been typically classified based on a summed score of ≥16 [23–25].

We also considered a selection of possible confounders in the relationship between MDD symptoms and barriers to care, including: age at baseline (per 10-year increase); sex (male vs. female); white ethnicity (yes vs. no); calendar year (per one-year increase); HIV serostatus (positive vs. negative); homelessness (yes vs. no); any injection drug use (yes vs. no); daily heroin use (yes vs. no); daily cocaine use (yes vs. no); daily crystal methamphetamine use (yes vs. no); daily prescription opioid use (yes vs. no); incarceration (yes vs. no); enrollment in opioid agonist therapy (yes vs. no); hospitalized (yes vs. no); sex work (yes vs. no); stable employment (yes vs. no); history of childhood physical abuse (yes vs. no); history of childhood sexual abuse (yes vs. no); and victim of violence (yes vs. no). With the exception of age, sex, calendar year, HIV serostatus, and history of childhood physical or sexual abuse, all variables were considered as time-updated variables of events reported in the six months prior to interview.

### Statistical analyses

As a first step, we examined descriptive and socio-demographic characteristics of the sample, stratified by having had recently experienced barriers to accessing health services at baseline. Comparisons were made using the Pearson’s χ2 test for binary variables and the Mann-Whitney U test for continuous variables. Next, we used bivariate logistic regression to estimate the relationship between the outcome (i.e., barriers to accessing health services in the last six months) and all explanatory variables, including CES-D summed score. Then, a multiple logistic regression model was constructed where all secondary variables significant at *p* < 0.10 in bivariate analyses were included. Of note, the explanatory variable and the primary outcome of interest covered the previous two weeks and six months, respectively; thus, the CES-D variable was lagged in response to potential temporal concerns.

In a stepwise manner, we compared the value of the coefficient in the full model to the value of the coefficient for the main relationship in each of the reduced models, the variable associated with the smallest relative change was dropped. We continued this iterative process until the maximum change exceeded 5%. Multicollinearity was assessed using the variance inflation factor. Finally, the following variables were forced back into the final model as these variables are known confounders in the main relationship: sex; homelessness; and injection drug use in the last six months.

As a secondary analysis, we accessed frequencies to determine common barriers to accessing health services among participants who reported ‘yes’ to the main outcome: in total and stratified by CES-D summed score (≥ 16 vs. < 16). All analyses were performed in R version 3.2.4 (Foundation for Statistical Computing, Vienna, Austria).

## Results

Baseline descriptive and socio-demographic characteristics, stratified by the outcome are presented in Table 1. Among a total of 1529 PWUD, 801 (87.3%) of VIDUS participants and 481 (79.1%) of ACCESS participants reported injecting drug use in the past six months. 415 (27.1%) reported barriers to accessing health services and 956 (62.5%) had a CES-D total score ≥ 16 at baseline. Among those with a CES-D total score ≥ 16 at baseline, 297 (71.6%) reported barriers to accessing health services whereas 650 (59.1%) reported no barriers to accessing health services, resulting in a difference of 12.5%. Five hundred twenty-one (34.1%) were female, 878 (57.4%) had white ancestry, and the median age at baseline was 43 years (quartile [Q]1 - Q3: 36–49 years). The median difference between the lagged date for the explanatory variable and current date for the outcome variable was 6 months (Q1 - Q3: 6-6).

**Table 1:** Baseline characteristics stratified by having had recently experienced barriers to accessing health services among people who use illicit drugs in Vancouver, Canada (*N* = 1529)

	Total *n* = 1529, N (%)	Barriers to accessing health services in the last six months		*p –* value
		Yes *n* = 415, *N* (%)	No *n* = 1099, *N* (%)	
Characteristic				
CES-D total score				
≥ 16	956 (62.5)	297 (71.6)	650 (59.1)	< 0.001
< 16	459 (30)	89 (21.4)	364 (33.1)	
Age^a^				
median	43.0	42.4	43.1	0.119
IQR	(36.2–48.6)	(35.0–47.8)	(36.6–49.0)	
Gender				
male	1008 (65.9)	258 (62.2)	739 (67.2)	0.063
female	521 (34.1)	157 (37.8)	360 (32.8)	
Caucasian ethnicity				
yes	878 (57.4)	248 (59.8)	624 (56.8)	0.295
no	651 (42.6)	167 (40.2)	475 (43.2)	
Calendar year				
median	2007	2007	2007	0.776
IQR	(2006–2009)	(2006–2009)	(2006–2009)	
HIV serostatus				
positive	600 (39.2)	147 (35.4)	445 (40.5)	0.071
negative	929 (60.8)	268 (64.6)	654 (59.5)	
Homelessness^b^				
yes	481 (31.5)	164 (39.5)	311 (28.3)	< 0.001
no	1041 (68.1)	246 (59.3)	786 (71.5)	
Any injection drug use^b^				
yes	1282 (83.8)	378 (91.1)	891 (81.1)	< 0.001
no	244 (16)	37 (8.9)	206 (18.7)	
Daily heroin use^b^				
yes	346 (22.6)	107 (25.8)	234 (21.3)	0.066
no	1179 (77.1)	308 (74.2)	862 (78.4)	
Daily cocaine use^b^				
yes	130 (8.5)	49 (11.8)	81 (7.4)	0.006
no	1393 (91.1)	365 (88.0)	1014 (92.3)	
Daily crystal methamphetamine use^b^				
yes	74 (4.8)	28 (6.7)	44 (4.0)	0.026
no	1450 (94.8)	387 (93.3)	1051 (95.6)	
Daily prescription opioid use^b^				
yes	97 (6.3)	39 (9.4)	58 (5.3)	0.004
no	1429 (93.5)	376 (90.6)	1039 (94.5)	
Incarceration^b^				
yes	228 (14.9)	72 (17.3)	153 (13.9)	0.084
no	1298 (84.9)	340 (81.9)	946 (86.1)	
Enrollment in opioid agonist therapy^b^				
yes	667 (43.6)	181 (43.6)	481 (43.8)	0.994
no	858 (56.1)	232 (55.9)	616 (56.1)	
Hospitalized^b^				
yes	302 (19.8)	91 (21.9)	208 (18.9)	0.191
no	1227 (80.2)	324 (78.1)	891 (81.1)	
Sex work^b^				
yes	222 (14.5)	71 (17.1)	148 (13.5)	0.064
no	1300 (85.0)	340 (81.9)	948 (86.3)	
Stable employment^b^				
yes	371 (24.3)	94 (22.7)	275 (25.0)	0.338
no	1158 (75.7)	321 (77.3)	824 (75.0)	
History of childhood physical abuse				
yes	1061 (69.4)	296 (71.3)	757 (68.9)	0.076
no	410 (26.8)	95 (22.9)	309 (28.1)	
History of childhood sexual abuse				
yes	655 (42.8)	207 (49.9)	444 (40.4)	< 0.001
no	824 (53.9)	192 (46.3)	621 (56.5)	
Victim of violence^b^				
yes	324 (21.2)	125 (30.1)	195 (17.7)	< 0.001
no	1200 (78.5)	288 (69.4)	901 (82.0)	

^a^Per 10-year increase

^b^Activities reported in the six months prior to interview

As presented in Table 2, in a multiple logistic regression analyses after adjusting for a range of confounders, lagged CES-D total score ≥ 16 remained independently associated with barriers to accessing health services (adjusted odds ratio [AOR] = 1.40; 95% confidence interval [CI]: 1.03–1.92). The relative risk (RR) was 1.20 (95% CI, 1.12–1.29). Suffered physical violence also remained independently associated with barriers to accessing health services (AOR = 1.38; 95% CI: 1.01–1.86). Among those who reported a CES-D total score of ≥16 and barriers to accessing health services, the most commonly reported barriers included: long wait lists/times (38.1%); treated poorly by health care professionals (30.0%); and difficulty keeping appointments (18.5%). Among those who reported a CES-D total score <  16 and barriers to accessing health services, the most commonly reported barriers included: long wait lists/times (30.3%); treated poorly by health care professionals (27.0%); and difficulty keeping appointments (14.6%).

**Table 2:** Multiple logistic regression model to determine the relationship between CES-D score and barriers to accessing health services among people who use illicit drugs in Vancouver, Canada (*n* = 1529)

	Unadjusted		Adjusted	
Characteristic	Odds ratio (95% CI)	*p -* value	Odds ratio (95% CI)	*p -* value
CES-D total score^a^				
(≥ 16 vs. < 16)	1.63 (1.23–2.18)	0.001	1.40 (1.03–1.92)	0.035
Age				
(per 10-year increase)	0.81 (0.70–0.93)	0.002	0.88 (0.76–1.02)	0.096
Gender				
(male vs. female)	0.85 (0.66–1.10)	0.214	0.99 (0.74–1.31)	0.927
Caucasian ethnicity				
(yes vs. no)	1.02 (0.80–1.31)	0.869		
Calendar year				
(per 1-year increase)	0.99 (0.94–1.03)	0.564		
HIV serostatus				
(positive vs. negative)	0.82 (0.64–1.06)	0.139		
Homelessness^b^				
(yes vs. no)	1.31 (1.00–1.72)	0.049	1.11 (0.83–1.49)	0.467
Any injection drug use^b^				
(yes vs. no)	1.37 (1.03–1.84)	0.032	1.21 (0.89–1.65)	0.224
Daily heroin use^b^				
(yes vs. no)	1.13 (0.84–1.52)	0.419		
Daily cocaine use^b^				
(yes vs. no)	0.87 (0.53–1.35)	0.543		
Daily crystal methamphetamine use^b^				
(yes vs. no)	1.80 (1.05–3.01)	0.027		
Daily prescription opioid use^b^				
(yes vs. no)	1.44 (0.87–2.32)	0.140		
Incarceration^b^				
(yes vs. no)	1.72 (1.22–2.41)	0.002		
Enrollment in opioid agonist therapy^b^				
(yes vs. no)	0.92 (0.72–1.18)	0.511		
Hospitalized^b^				
(yes vs. no)	1.67 (1.22–2.27)	0.001		
Sex work^b^				
(yes vs. no)	1.08 (0.74–1.54)	0.689		
Stable employment^b^				
(yes vs. no)	0.81 (0.59–1.10)	0.190		
History of childhood physical abuse				
(yes vs. no)	1.47 (1.09–1.99)	0.012		
History of childhood sexual abuse				
(yes vs. no)	1.44 (1.12–1.86)	0.004	1.30 (0.99–1.70)	0.060
Victim of violence^b^				
(yes vs. no)	1.55 (1.16–2.07)	0.003	1.38 (1.01–1.86)	0.039

*CES-D* Center for Epidemiological Studies Depression Scale, *CI* Confidence Interval

^a^CES-D variable is lagged

^b^Activities reported in the six months prior to interview

## Discussion

In the present study, more than half of PWUD showed MDD symptoms. We observed a high proportion of participants who reported barriers to accessing health services. We also found a positive and independent relationship between MDD and barriers to accessing health services among PWUD, after adjusting for various confounders.

While these results corroborate the findings from previous research in that we found a high prevalence of barriers to care among PWUD [19, 20, 26], there are some inconsistencies between our results and previous findings, which are likely attributable to differences in study environments. For example, in a study conducted in the United States (US), the sample was comprised of 393 patients who met criteria for both MDD and SUD (i.e., 100% prevalence of MDD-SUD co-occurrence) [20]. Our study sample was comprised of 1529 PWUD, 573 (37.5%) of which reported no MDD symptoms. Furthermore, this US-based study focused primarily on the role of financial barriers to accessing SUD and evidence-based MDD care. Our study provides new insight into client perspectives in a setting where there are less financial barriers to SUD or evidence-based MDD care due to universal health care. That being said, it is important to consider that British Columbia’s provincial health insurance program covers limited mental health care.

In line with previous findings, we found a relationship between MDD and barriers to care among PWUD [20]. Encouragingly, MDD symptoms in PWUD often improve following SUD treatment initiation [5, 27]. Given the central role of primary care physicians in SUD care in Canada, and abroad [13, 28], it is likely that the integration of SUD care into primary care settings would lead to improved depressive symptoms for many. In circumstances where MDD is not a self-limited condition, integration of evidence-based MDD care with primary care could also potentially reduce wait times, improve continuity of care, and increase patient satisfaction [29, 30]. Research data suggest scaling-up collaborative and integrated care pathways as another potential approach to reduce wait times while improving continuity and satisfaction with care among individuals with SUD-MDD co-occurrence [31, 32].

The perception of being ‘treated poorly by health care professionals’ may be a consequence of mental illness-related stigma, substance use-related stigma, and/or abstinence-only based policies within healthcare settings. Our findings underscore a widely prevalent perception of poor treatment by health care professionals among PWUD, regardless of the presence of MDD symptoms [33]. Empirical evidence suggests that a certain level of “mutual mistrust” can exist between providers and PWUD. This may be partially attributable to the fact that providers who are not specialized in addiction medicine lack confidence in providing care for those living with SUD. This can lead to inconsistency in care or avoidance by the provider, which could be interpreted as a sign of maltreatment by PWUD [34]. Provider training such as addiction medicine programs which have sought to integrate substance use care with the rest of medicine, anti-stigma interventions, contact-based education approaches which enhance provider comfort and skills in treating those with MDD and/or SUD, and efforts to abolish abstinence-only based policies in healthcare settings may help to address this barrier [30, 35]. Future research should explore the impact of these diverse and innovative strategies to improve MDD and SUD diagnosis, uptake and continuity of care among PWUD.

Our findings should be viewed in the context of several limitations. First, measurement of the primary explanatory variable and the outcome were reliant on self-reported data, which is susceptible to social desirability and recall bias. Second, as with all MDD screening measures, there are psychometric limitations of the CES-D [36, 37]. However, the CES-D has demonstrated validity and reliability in numerous populations (including among PWUD), and has been used extensively in epidemiologic research [38–40]. Third, we relied on a lagged explanatory variable; therefore, there may have been a time gap between when the explanatory and outcome variables were measured. However, we found that the median time between these variables was 6 months. Given that the median duration of an MDD episode is approximately 20 weeks, we decided to use a lagged explanatory variable in our analyses. Forth, despite adjusting models for known confounders, results may be subject to underlying residual and unmeasured confounding. Fifth, these data are observational, thus we were unable to draw conclusions regarding causality. Finally, participants were not randomly selected and therefore, the extent to which these results may be generalized beyond our setting is unclear.

## Conclusion

Overall, our findings demonstrate an essential need for scaling-up access to evidence-based SUD and MDD care among PWUD populations. The integration of evidence-based SUD and MDD care with primary care, scaling-up collaborative and integrated care pathways (to diminish long wait lists/times), provider education, anti-stigma interventions, and efforts to extinguish abstinence-only based policies in healthcare settings (to diminish perceived stigma by health care professionals) may help to alleviate MDD and/or SUD symptoms among PWUD populations. Although future studies are required to fully elucidate the impact of SUD-MDD co-occurrence and to provide guidance for healthcare providers facing the challenge of treating SUD-MDD co-occurrence, the results of this analysis clearly indicate that policies and interventions tailored to address these barriers are urgently needed to mitigate the alarming rates of morbidity and mortality experienced among individuals with SUD-MDD co-occurrence.

## Abbreviations

ACCESS: AIDS Care Cohort to evaluate Exposure to Survival Services
AOR: Adjusted odds ratio
CES-D: Centre for Epidemiologic Studies Depression
CI: Confidence interval
GAD: Generalized anxiety disorder
MDD: Major depressive disorder
PHN: Personal Health Number
PTSD: Posttraumatic stress disorder
PWUD: People who use illicit drugs
Q: Quartile
US: United States
VIDUS: Vancouver Injection Drug Users Study
